# Supplementary material for: TEITbase: a database for transposable element (TE)-initiated transcripts in human cancers
Source: Database (Oxford). 2026 May 19;2026:baag025. doi: 10.1093/database/baag025 (PMC13184619; doi:10.1093/database/baag025)
Supplement: baag025_Supplemental_Files [file baag025_supplemental_files.zip › Supplementary_Information-0412.pdf]

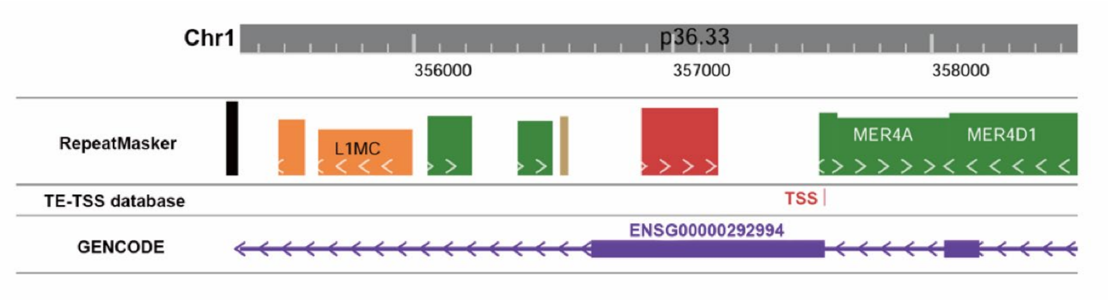

Fig. S1. An example of TE exonization in the TE-TSS database.

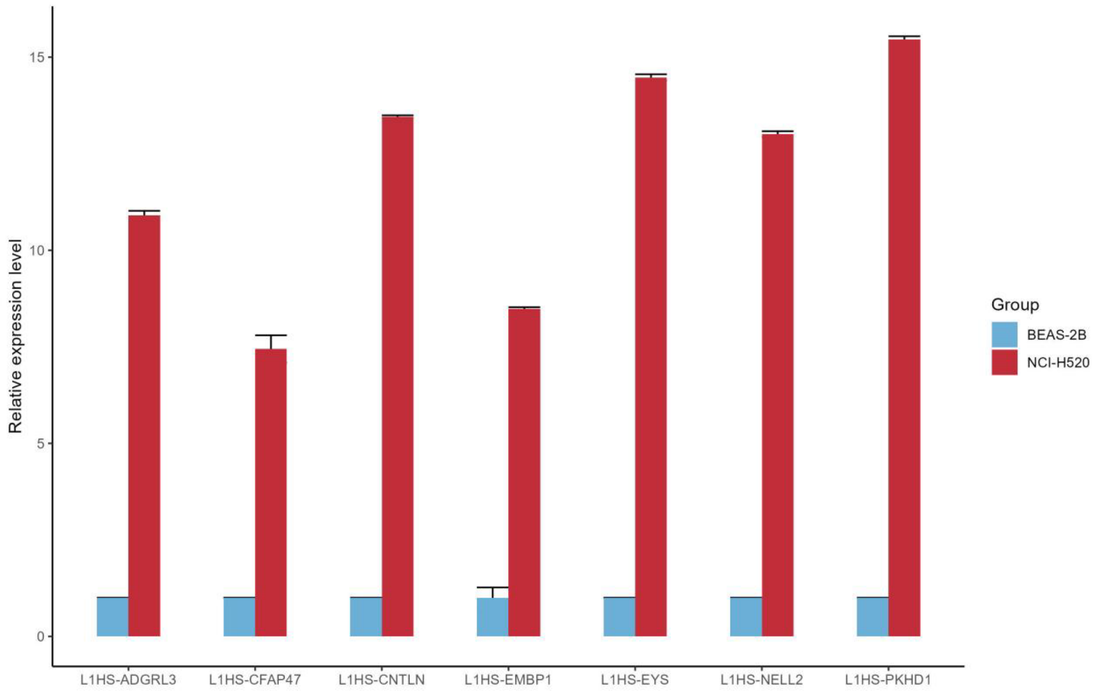

Fig. S2. RT-qPCR validation of TE-initiated transcripts with high tumor-specific scores.

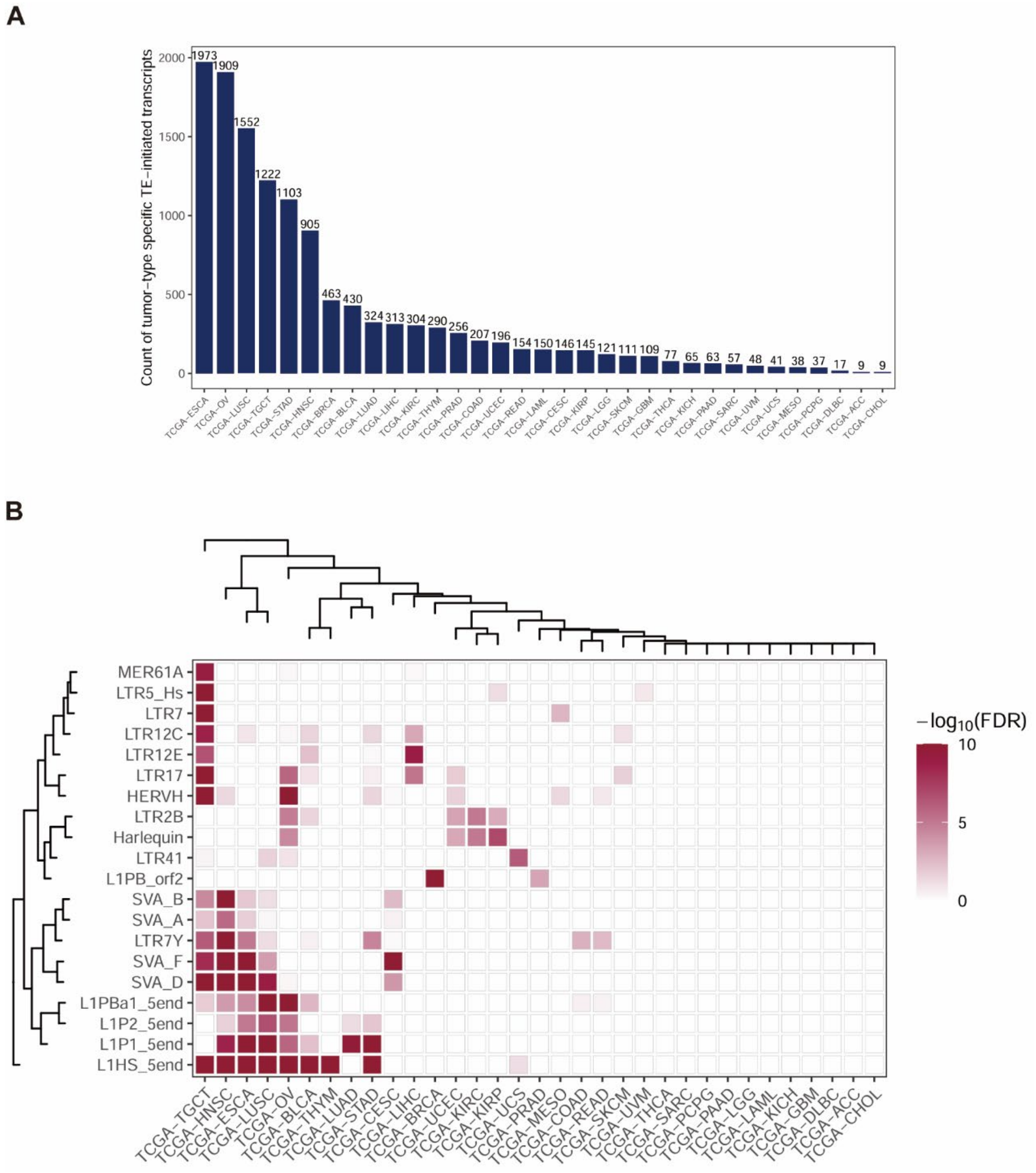

Fig. S3. (A) The count of preferentially expressed TE-initiated transcripts across various tumor types. (B) The enrichment analysis of TE families for TE-initiated transcripts across various tumor types is displayed using color intensities corresponding to their -$\log_{10}$(FDR) values.
